# Associations between parental well-being and early learning at home before and during the COVID-19 pandemic: observations from the China Family Panel Studies

**DOI:** 10.3389/fpsyg.2023.1163009

**Published:** 2023-05-15

**Authors:** Shuyang Dong, Nirmala Rao

**Affiliations:** Faculty of Education, The University of Hong Kong, Pokfulam, Hong Kong SAR, China

**Keywords:** COVID-19, educational investment, early childhood education and care (ECEC), early learning, parental well-being, parental health

## Abstract

**Background:**

COVID-19-related lockdowns and preschool closures resulted in many young children spending all their time at home. Some parents had to manage child care while working from home, and increased demands may have led them to experience considerable stress. Evidence indicates that among parents with young children, those who had pre-existing mental and physical conditions adapted less well than other parents. We considered associations between parental well-being and the home learning environment for young children.

**Method:**

We leveraged data from the nationally representative China Family Panel Studies. We analyzed longitudinal data collected before (2018) and during (2020) the pandemic. Participants were parents of 1,155 preschoolers (aged 3–5 years in 2020). Moderated mediation models were conducted. Maternal and paternal psychological well-being, depression, physical health, and physical illness in 2018 and 2020 were predictors. The frequency of marital and intergenerational conflicts in 2020 were mediators. Primary caregiver-reported engagement in home learning activities and family educational expenditure and parent-reported time spent on child care in 2020 were outcome variables. The number of COVID-19 cases in each province 3 months before the 2020 assessment was the moderator. Child, parental, and household characteristics and urbanicity were covariates.

**Results:**

Controlling for covariates, improvements in parental psychological well-being predicted more home learning activities and increases in paternal depression predicted less time spent by fathers on child care. Negative changes in maternal physical health predicted less family educational expenditure and mothers spending more time on child care. Family conflicts mediated the association between maternal physical illness in 2018 and family educational expenditure. The number of COVID-19 cases in a province (i) was positively associated with mothers spending more time on child care, (ii) moderated the association of improvements in maternal physical health and mothers spending less time on child care, and (iii) moderated the association of family conflicts and more family educational expenditure.

**Conclusion:**

The findings indicate that decreased parental psychological and physical well-being foretells reductions in monetary and non-monetary investment in early learning and care at home. Regional pandemic risk undermines maternal investment in early learning and care, especially for those with pre-existing physical conditions.

## 1. Introduction

COVID-19 confinement measures such as lockdowns, quarantines, and school closures have resulted in billions of children spending all their time at home (Gromada et al., 2020). The timing and length of school closures varied based on actual and predicted peaks and troughs of the pandemic and across countries. The extent of children’s learning losses varied depending on family characteristics, parental and school support for learning, internet connectivity, and where children lived (Yoshikawa et al., 2020; McCoy et al., 2021; Rao and Fisher, 2021). Furthermore, adults’ physical health and psychological well-being deteriorated immediately after the implementation of containment strategies associated with this unprecedented global crisis (Wang et al., 2020; Robinson et al., 2022). Within adult populations, parents were adversely affected by the changes in daily routines and socioeconomic hardships amid the pandemic. This is because the new norm of homeschooling and family-based care necessitated parents managing child care while working from home with little preparation. Therefore, most parents experienced considerable stress (Gromada et al., 2020; Prime et al., 2020).

During the pandemic, parents with young children reported even poorer health and well-being outcomes than parents with school-aged children and adolescents (Huebener et al., 2021; Westrupp et al., 2023). Studies have linked parental psychological and physical well-being with parental provisions of home learning activities and educational investment in young children (e.g., Peacock-Chambers et al., 2017; Nuttall et al., 2019). Thus, there are concerns about the extent to which parents of young children can provide nurturing care and promote early learning at home without necessary social support. Lack of access to child care and early childhood education services may adversely impact parents with pre-existing mental and physical conditions in particular. Without center-based services, parents with existing psychological and physical conditions may not be able to provide a nurturing and stimulating home learning environment for their children (Prime et al., 2020; Rao and Fisher, 2021).

Thus far, there remains a paucity of studies that have examined changes in health and well-being among Chinese parents with young children during the pandemic and how such pandemic-related parental changes potentially predict parental investment in promoting children’s early learning at home. To address these research gaps, the current study leverages data from a nationally representative sample of Chinese mothers and fathers with young children to investigate these critical questions. Rather than situating the study in the context of the initial outbreak of COVID-19, we focus on parental well-being and functioning in the latter, longer situation experienced by Chinese families when there was a low transmission rate of COVID-19 at the country level but non-negligible variations in the pandemic risks at the province level. Therefore, the findings have the potential to advance knowledge of what factors predict the exertion of effective strategies to promote young children’s learning amid the pandemic. The findings can further suggest educational and psychological strategies that address the needs of both parents and young children during the homeschooling phase.

### 1.1. Parental psychological and physical well-being during the pandemic

Following Brock and Laifer (2020) and Prime et al. (2020), we posit that disruptions caused by the pandemic, such as reduced financial security, compulsory social distancing, and home confinement, may lead to the decreased psychological well-being of parents. Indeed, systematic reviews and meta-analyses have confirmed that parents experienced heightened distress, stress, anxiety, and depression during the pandemic (Fong and Iarocci, 2020; Lateef et al., 2021). Parents often exhibit greater levels of psychological problems relative to adults without children (Elder and Greene, 2021; Huebener et al., 2021; Lateef et al., 2021). Within a constellation of pandemic-induced psychological problems, the emergence of depressive symptoms is notable because these symptoms have markedly increased and persisted across the pandemic (Robinson et al., 2022).

Existing (yet scarce) evidence supports an increase in parental depression from the pre-pandemic to pandemic times (Galbally et al., 2022; Westrupp et al., 2023). In a similar vein, preliminary evidence drawn from Chinese families indicates that parents experienced more parenting stress during the pandemic than before (Zhang et al., 2021). Parenting stress is strongly associated with parental depression because stress stemming from fulfilling various parental responsibilities tended to arise and stack up amid the pandemic, which can result in prolonged negative moods and unresolvable distress in parents (Johnson et al., 2022; Babore et al., 2023). However, as far as we know, no studies have paid particular attention to the changes in the depression of Chinese parents with young children. It is possible that these Chinese parents have displayed more depressive symptoms during the pandemic in comparison with before the pandemic.

In addition to parental depression, the pandemic has also affected other (yet under-documented) aspects of psychological well-being in parents, such as life satisfaction and confidence in the future. Decreased life satisfaction among parents with young children has been reported. For instance, before the pandemic, German parents with young children had the highest life satisfaction within parent populations. During the pandemic, however, the life satisfaction of these parents significantly declined, and they reported the lowest satisfaction with child care (Huebener et al., 2021). Moreover, the levels of parental quality of life decreased from the pre-pandemic to pandemic times (Rohde et al., 2022). Again, worries and stress related to the COVID-19 pandemic and parental responsibilities, which already compound parental mental health, may undermine positive parental evaluations of life and result in pessimism about the future (Limbers et al., 2020; Taha et al., 2022).

Further to Brock and Laifer (2020) and Prime et al. (2020), whose theories mainly concern investigating family functioning through parental psychological responses to the pandemic, increasingly evident in the literature is the co-occurrence of somatic symptoms with individuals’ deteriorating psychological states. Unsurprisingly, parents have reported considerable sleep disturbance and reductions in everyday activity levels across the lockdown and quarantine phases (Elder and Greene, 2021; Hernández-Jaña et al., 2022). The confinement measures first remove opportunities to actively work out in public spaces or passively participate in physical activities such as commuting to work (Dai et al., 2021; Hernández-Jaña et al., 2022). Sleep problems such as a shortened sleep duration and postponed bedtimes may subsequently emerge as accompanying symptoms (Majumdar et al., 2020).

We argue that the pandemic has had holistic, all-embracing impacts on most, if not all, aspects of parental well-being. It is critical to examine processes that affect both psychological responses and physical well-being to understand better how parents behave and function in uncharacteristic times. Correlations between physical health and physical illness have been established for both mental health problems (Majumdar et al., 2020; Wang et al., 2020) and positive psychological indicators (Limbers et al., 2020; Dai et al., 2021). For example, physical health and the frequency of participation in physical activity were associated with a more positive evaluation of one’s well-being and better emotion regulation (Dai et al., 2021). Participation in physical activity was related to better quality of life in the social relationship domain (Limbers et al., 2020). In contrast, sleep quality was negatively associated with depression during the pandemic (Grey et al., 2020). In the long run, sleep disturbances and physical inactivity may be translated into the physical symptom load, characterized by overall physical health being worse off and a greater burden of out-patient and in-patient health care (Beutel et al., 2019).

In brief, existing studies have shown that parental psychological and physical well-being was likely threatened by the COVID-19 pandemic and its relevant confinement measures. The triggers of such detrimental impacts include, but are not limited to, pandemic-related stress and worries, pandemic-related regulations that limit one’s agency and mobility, and child care and homeschooling responsibilities that conflict with work arrangements. Importantly, these stressors have all-embracing influences on parental well-being. That is, they elicit unpleasant psychological responses and amplify the physical symptom load.

### 1.2. Psychological and physical well-being with provisions of home-based early learning

Of developmental relevance to young children is that compromised psychological and physical well-being hampers parents’ abilities to provide nurturing care and quality learning activities at home (Fong and Iarocci, 2020; Prime et al., 2020). Center-based early childhood education and care and schools were closed to contain the spread of the coronavirus in many countries. Providing stimulating home learning activities and investing in child education and care at home became vital for young children’s early learning amid the pandemic (Yoshikawa et al., 2020). In China, online courses were delivered for primary and secondary school students from the spring of 2020 onwards to compensate for potential losses of educational attainment (Wang et al., 2020). Preschoolers, however, were not required to participate in any mandatory online educational programs, thus precluding their access to alternative learning opportunities during the COVID-19 pandemic (Dong et al., 2020). This means that parents or other domestic caregivers were responsible for educating and caring for young children.

A growing body of research has focused on associations between parental psychological and physical well-being and parental provisions of early learning and care at home during the pandemic. Among Chinese parents with young children, parental pandemic-related stress is negatively associated with the frequency of providing various learning activities at home (i.e., literacy, math, and motor activities) (Zhang et al., 2021). In contrast, family physical health is positively related to parental involvement in early learning activities at home (Zhang, 2022). Regarding parents from other countries, depression relates negatively to parental perceived preparation to educate at home (Lee et al., 2021), and parental stress impedes the provision of home learning activities (Oppermann et al., 2021). In contrast, self-confidence in adapting to the pandemic is positively associated with parental involvement in formal homeschooling and informal learning activities (Treviño et al., 2021).

In brief, parental psychological and physical well-being may be associated with parental provisions of early learning and care at home during the pandemic. However, we identified several limitations in these previous studies. First, past studies have seldom considered how *changes* in parental well-being with the pandemic predict parental provisions of early learning and care at home. Second, no studies have included and examined how positive *and* negative parental psychological *and* physical well-being indicators are associated with parental provisions of early learning and care at home. Third, the existing studies have not considered all three facets of monetary and non-monetary educational investment: family educational expenditure, quality educational activities, and time spent on education and care (Bianchi et al., 2004, pp. 190–191). Therefore, this study addresses the above-mentioned limitations and provides nuanced knowledge about how parental psychological and physical well-being influenced the early learning support and care children received at home during the COVID-19 pandemic.

### 1.3. The present study

The primary aims of this study are (i) to compare the psychological and physical well-being of Chinese parents with young children before and during the pandemic, and (ii) to elucidate how changes in parental well-being are predictive of parental provisions of early learning activities and care at home. To this end, we leveraged the most recent longitudinal data from the China Family Panel Studies (CFPS; Xie and Hu, 2014). In the face of the unexpected COVID-19 pandemic, the CFPS research team adapted their assessment methods by integrating new technologies for interviews and continued to carry out the scheduled biennial survey with the original sample in 2020. This allowed the collection of repeated measures of parental well-being with a large sample of Chinese families from pre-pandemic to pandemic times. This also enabled us to answer the call in the field for comprehensive, longitudinally tracked investigations on caregivers’ health and family functioning throughout the pandemic (Brock and Laifer, 2020; Roubinov et al., 2020; Yoshikawa et al., 2020).

#### 1.3.1. Mediation of family conflicts

Further to these primary aims, we concur with Roubinov et al. (2020) on the complexity of pandemic-induced consequences and acknowledge the importance of the Prime et al. (2020) whole-family-function model for understanding children’s early learning experiences during the pandemic. Therefore, we examined the mediating roles that intergenerational conflicts (between parents and grandparents) and marital conflicts possibly play in the associations between parental well-being and parental provisions of early learning and care at home. In the Prime et al. (2020) model, the marital system, parent-grandparent system, and parent-child system are all directly influenced by parental psychological well-being. One of the hypothesized mechanisms is that pre-existing family vulnerabilities, including parental mental health problems and physical symptom load, exacerbate the risk of family conflicts and poor relationships during the pandemic. This risk may, in turn, hinder parental functioning and parents may show decreases in engagement in educational activities and spend less time on child care (Prime et al., 2020; Rao and Fisher, 2021).

Empirical evidence has indicated the possibilities of the mediating effects of intergenerational and marital conflicts. For instance, family conflicts are associated with parental stress and fewer home learning activities (Oppermann et al., 2021). While family conflicts adversely impacted maternal mental health in Italy, the Netherlands, and China, only in China could support from grandparents buffer these negative influences on maternal mental health (Guo et al., 2022). Moreover, for marital conflicts, those with less spousal support are more likely to experience parenting stress and verbal marital conflict (Chung et al., 2022). From the perspective of parental lived experiences, marital conflicts and homeschooling difficulties were reported by parents who experienced psychological distress during the pandemic (Weaver and Swank, 2021). Nevertheless, the quantitative analyses revealed that marital conflicts did not predict changes in home learning activities (Oppermann et al., 2021).

The inconsistency of results concerning marital conflicts should be discussed within the theme of gender disparities in offering home-based child care amid the pandemic. Studies have consistently shown that, for parents with young children, mothers are at higher risk than fathers for unemployment owing to school closures and an unequal division of family care responsibilities (Petts et al., 2021; Yavorsky et al., 2021). Relative to working fathers, working mothers with young children spend considerably more time in educational activities, caregiving, and working while providing child care (Yavorsky et al., 2021; Augustine and Prickett, 2022). Thus, it seems reasonable to expect that the extent to which mothers experience pandemic-related stress would differ from that of fathers. Mothers would be particularly at high risk for deteriorating psychological and physical well-being and poor interpersonal relationships. These factors put mothers, but not necessarily fathers, in an unfavorable situation where providing home-based learning activities and child care is especially demanding and exhausting. To understand such a “gendered pandemic” phenomenon, we conducted statistical models separately for mothers and fathers to unveil possibly differential gender patterns in the processes of interest.

#### 1.3.2. Moderation of regional pandemic risk

Last, following Brock and Laifer (2020), we postulate that COVID-19-related factors, such as the transmission rates of COVID-19 in different regions, might modulate perceived threat and stress levels. The extent to which parental well-being changes and the urgent need for home-based education and child care should be understood in light of how parents from various regions encounter varying transmission risks of COVID-19. This is because parents from different regions may experience different levels of pandemic-related stress.

Indeed, preliminary evidence has suggested regional differences in the Chinese general populations’ mental health outcomes. People from provinces at higher risk of directly or indirectly contracting confirmed cases report more psychological problems than those from other provinces (Ren et al., 2020). Swiss parents who lived in regions with higher pandemic risks worried about their children’s health more than parents from other regions (Seiler et al., 2021). To extend this body of research, we further examine if regional pandemic risk directly affects parental provisions of early learning and care at home and if regional pandemic risk moderates the links between parental well-being and early learning and care provisions.

## 2. Materials and methods

### 2.1. Participants

Data from the CFPS (Xie and Hu, 2014) that were collected before (2018) and during (2020) the COVID-19 pandemic were analyzed. The CFPS, a biennially conducted project from 2010, used a multistage probability sampling strategy to recruit a nearly nationally representative sample of Chinese families from 25 provinces. In each wave, information about participants’ social, economic, educational, and health outcomes was collected together with information at the community and family levels. Most participants were interviewed in person in 2018, whereas most were interviewed using the computer-assisted technique in 2020, owing to the relatively strict regulations for domestic travel.

We limited our sample to parents of 1,155 preschoolers (aged 3–5 years) at the 2020 assessment. We extracted data from the 2018 assessment on their prior psychological and physical well-being and demographics. Across the two assessments, most families were assessed in July and August (79.9% in 2018 and 85.2% in 2020). In 2020, all the interviews were conducted from July to December, during which the transmission risk of COVID-19 was relatively low overall in China though regional pandemic risks varied considerably. Demographic information on the included families is presented in Table 1.

**Table 1:** Demographic information of the participants.

	2018			2020		
	*N*	*M*/%	Range	*N*	*M*/%	Range
Child gender	1,155			1,155		
Boys (%)	535	53.7		535	53.7	
Girls (%)	620	46.3		620	46.3	
Child age in years	1,155	1.94	1–3	1,155	3.94	3–5
Mother age in years	934	29.65	17–44	688	31.83	20–46
Mother education level in years	894	10.83	0–19			
Mother annual income (Yuan)	661	18487.26	0–500,000	666	23857.52	0–255,000
Mother career prestige score	549	43.53	20–88			
Father age in years	845	31.66	20–58	611	33.67	22–55
Father education level in years	847	10.93	0–22			
Father annual income (Yuan)	579	43919.40	0–840,000	592	51114.42	0–400,000
Father career prestige score	567	41.36	20–88			
Urbanicity	1,155			1,155		
Urban areas (%)	554	48.0		479	41.5	
Rural areas (%)	584	50.6		530	45.9	
Missing (%)	17	1.5		146	12.6	
Family size	1,155	5.47	1–21	946	5.41	1–15

### 2.2. Measures

#### 2.2.1. Parental psychological and physical well-being in 2018 and 2020

##### 2.2.1.1. Negative indicator of psychological well-being: depression

Maternal and paternal depressive symptoms were measured using the Center for Epidemiologic Studies Depression Scale (CES-D; Radloff, 1977; He et al., 2013). The CES-D is rated on a 4-point Likert-type scale ranging from 0 (rarely or none of the time/less than 1 day a week) to 3 (most or all of the time/5–7 days a week). This measure has been adapted to and widely used with Chinese populations (He et al., 2013). At the 2018 and 2020 assessments, an eight-item, short version of the CES-D was administered (CES-D8; e.g., “I felt depressed”). Open access to participants’ reports on individual items was not available due to the privacy protection protocol of the project. Nevertheless, using nationally representative samples, He et al. (2013) have confirmed that the CES-D8 is a reliable tool for Chinese populations, with Cronbach’s *α* = 0.85. Maternal and paternal *depression* was indexed by the sum scores of these eight items, with a larger score indicating more depressive symptoms.

##### 2.2.1.2. Positive indicators of psychological well-being: life satisfaction and faith in the future

Four items were used to index maternal and paternal psychological well-being, including “are you satisfied with your life,” “how confident are you about your future,” “are you happy,” and “do you think you are popular.” The first two items are rated on a 5-point Likert-type scale ranging from 1 (very unsatisfied/not confident at all) to 5 (very satisfied/very confident). The latter two items are rated on an 11-point scale ranging from 0 to 10. These items capture individuals’ evaluations of their life satisfaction, contentment, and confidence in the future. These positive cognitions have been examined in other family studies conducted amid the pandemic (e.g., Limbers et al., 2020; Huebener et al., 2021). With all the mean inter-item correlations being within the standard range of 0.15–0.50 (Clark and Watson, 1995), these four items showed good internal consistency of 0.36 for mothers in 2018, 0.33 for fathers in 2018, 0.41 for mothers in 2020, and 0.39 for fathers in 2020. Factor analyses, individually for maternal and paternal reports in 2018 and 2020, all yielded one factor, which explained about 50–56% of the total variance in these four items. The factor scores of *psychological well-being* were used, with a higher score indicating better psychological well-being.

##### 2.2.1.3. Negative indicators of physical well-being: health conditions, illness, and hospitalization

Four items were used to assess maternal and paternal physical symptom load, including “how would you rate your health status” on a 5-point scale from 1 (excellent) to 5 (poor), “how would you rate your current health status compared to a year ago” on a 3-point scale including 1 (better), 2 (no change), and 3 (worse), “during the past 6 months, have you had any doctor-diagnosed chronic disease” using binary options of 0 (no) and 1 (yes), and “in the past year, were you ever been hospitalized due to illness” using binary options of 0 (no) and 1 (yes). Despite the categorical nature of these rating scales, the internal consistency of these four items was acceptable according to the criterion (0.15 ~ 0.50; Clark and Watson, 1995): 0.16 for mothers in 2018, 0.19 for fathers in 2018, 0.15 for mothers in 2020, and 0.17 for fathers in 2020. Factor analyses yielded one or two factors, but the factor loadings of the four items were all positive and larger than 0.43 for the only factor or the first factor. Correspondingly, these factor scores, which explained about 37–40% of the variance in these four items and assessed parental *physical illness*, were used in the analyses. A higher physical illness score indicates a higher level of the physical symptom load.

##### 2.2.1.4. Positive indicators of physical well-being: exercise, sleep duration, and bedtime

Three items were used to index maternal and paternal health behaviors and healthy habits, including “what time do you usually go to bed at night,” “in general, how long do you sleep on weekdays/weekends,” and “how often and how long did you participate in physical exercise in the past week.” The instructions were worded in slightly different ways between the two assessments. Participants directly reported their frequency of exercise per week in 2018, whereas in 2020, participants were asked to choose from eight options that indicated different exercise frequencies. To make scores comparable across the two assessments, we weighted the length of weekdays and weekends for sleep duration and calculated, on average, how long the participant slept every day. For exercise duration, we transformed the eight options of exercise frequencies at the 2020 assessment into weekly frequencies and multiplied them with the exercise duration each time to approximate, on average, how long the participant participated in physical exercise per week. Maternal and paternal physical health was determined using the criteria: bedtime at 10 p.m. to midnight (Bliznak et al., 2019), sleep duration of 7–9 h (Consensus Conference Panel et al., 2015), and weekly exercise duration of 150–300 min (World Health Organization, 2020). Each index scored 1 point if participants’ data met the corresponding criterion. The sum score, indexing parental *physical health* and ranging from 0 to 3, was used.

#### 2.2.2. Outcomes: provisions of early learning and care at home in 2020

The child’s primary caregiver reported on three facets of home-based monetary and non-monetary investment in early learning and care (Bianchi et al., 2004). First, the caregiver answered the question, “in the past 12 months, how much did your family pay in total for the child’s education, including all kinds of payments to school and extracurricular education expenses.” This answer was used to index family *educational expenditure* in our study (see also Chen et al., 2023).

Second, the caregiver rated the frequencies of engagement in four early learning activities at home using a 5-point Likert-type scale ranging from 1 (several times a year or less than that) to 5 (every day). These were “read, for example, stories, to your child,” “buy books for your child, such as picture books,” “take the child out to play, such as strolling in the park, going to the playground, shopping, or having a picnic,” and “use toys, games, or other things to help the child learn characters.” These items have been confirmed to be reliable and valid for measuring parental provisions of early learning opportunities in China (e.g., Gong and Rao, 2023). In our study, the Cronbach’s α was 0.74. The mean frequency of engagement in these four learning activities indicates the provision of *home learning activities*.

Third, mothers and fathers responded to two questions individually: “how many hours do you spend taking care of the child every day” and “in general, how many times do you usually have dinner with your family (including eating outside) per week.” The answers to the first question indicate *child care time per day*, ranging from 0 to 24 (hours). The answers to the second question indicate how many days in a week the parent is usually with the child, ranging from 0 to 7 (days). We multiplied parental responses to the first and second questions to approximate *child care time per week*, which represents the total number of hours spent taking care of the child in a week. This latter outcome was included because compared with child care time per day, child care time per week is likely to tap the nature of continuous investment in child care and unlikely to be biased by parents recalling a special event (e.g., spending a whole day with the child due to an atypical event).

#### 2.2.3. Mediators: intergenerational conflicts and marital conflicts in 2020

The primary caregiver reported on the frequencies of intergenerational conflicts between parents and grandparents in the past 12 months concerning childrearing on a 5-point Likert-type scale ranging from 1 (never) to 5 (very often/5–7 times per week). This item is particularly relevant in the Chinese context where grandparents are likely to cohabitate with their children and take care of their grandchild when both parents go to work (Hoang and Kirby, 2020). The primary caregiver also reported on the frequencies of marital conflicts between mothers and fathers over childrearing in the past 12 months on a 5-point Likert-type scale ranging from 1 (never) to 5 (very often/5–7 times per week). These two items were used in a previous study that examined family conflicts in Chinese families with adolescents (Yang et al., 2022). *Intergenerational conflicts* and *marital conflicts* were treated as the mediators in our study.

#### 2.2.4. Moderator: regional pandemic risk in 2020

We assembled official reports on the number of COVID-19 cases in each province 3 months before the 2020 assessment. Across the 2020 assessment, the 6th and 7th editions of the Protocol for Prevention and Control of COVID-19 in China were enacted (National Health Commission of the People’s Republic of China, 2020; Zheng et al., 2020). Particularly noteworthy are the updated regulations for multi-method monitoring, close contacts management, and the scope and timing of administering confinement measures based on risk assessment results (Zheng et al., 2020).

After the enactment of these two protocols, the extent of social disruptions vis-à-vis the pandemic became quite distinct between regions. This is because varying strategies were administered to contain the spread of COVID-19 (e.g., in low-risk areas, monitoring people coming in from high-risk areas, while in high-risk areas, enacting lockdowns). The number of COVID-19 cases in each province 3 months before the 2020 assessment can approximate *regional pandemic risk* that reflected differing degrees of social disruptions.

#### 2.2.5. Covariates in 2018

Covariates, including child gender and age, maternal and paternal age, parents’ years of education, parents’ occupational prestige, family size, family income *per capita*, and urbanicity, were controlled for in the analyses. These factors are typically accounted for in the studies that examine how the pandemic influences family functioning (e.g., Robinson et al., 2022).

### 2.3. Analytic plan

**Figure 1:**
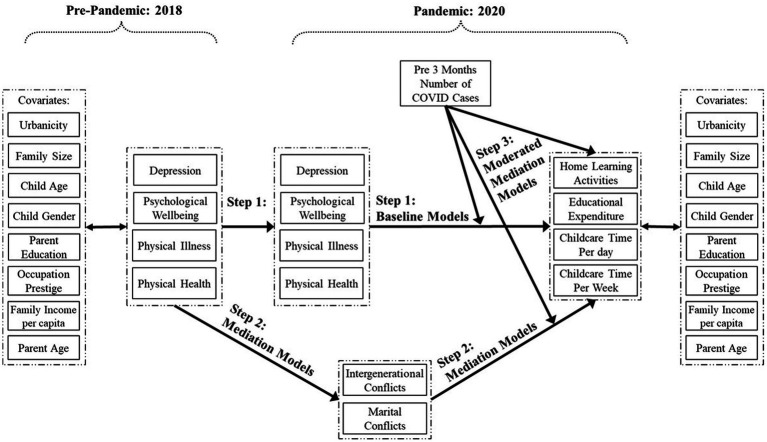
An overview of planned analyses.

First, we calculated the changes in maternal and paternal depression, psychological well-being, physical illness, and physical health before and during the pandemic. We next estimated how changes in parental well-being predict educational expenditure, home learning activities, and child care time per day and per week after accounting for the covariates. This baseline model (see step 1 in Figure 1) was conducted separately for maternal and paternal data. Then we conducted mediation analyses (step 2 in Figure 1). We entered intergenerational conflicts and marital conflicts to examine their mediating effects on the associations between parental well-being before the pandemic and the indicators of parental provisions of early learning and care. We also entered regional pandemic risk into the models to scrutinize its direct impacts on parental provisions of early learning and care. Finally, we conducted moderated mediation models (step 3 in Figure 1). In this step, we examined how regional pandemic risk moderates significant associations between changes in parental well-being and parental provisions of early learning and care and between family conflicts and parental provisions of early learning and care.

All the models were estimated in M*plus* using maximum likelihood with robust standard errors (MLR). The acceptance of model fit indices was determined using the following criterion: comparative fit index (CFI) > 0.90 in conjunction with the root mean square error of approximation (RMSEA) < 0.06 (Hu and Bentler, 1999). The missing data were handled by the full information maximum likelihood (FIML) method.

## 3. Results

### 3.1. Preliminary analyses

Means (*M*), standard deviations (*SD*), and correlations among variables are presented in Tables 2, 3. Regarding the outcome variables, educational expenditure, home learning activities, and parental child care time were not correlated, indicating that they represent distinctive facets of family investment in early learning and care amid the pandemic. For child care time specifically, mothers spent considerably more time than fathers in taking care of the child every day [paired *t*-test, *t*(355) = 16.27, *p* < 0.001, effect size, Hedges’ *g* = 1.18], and every week, [*t*(349) = 13.42, *p* < 0.001, *g* = 1.00]. For the mediators, intergenerational and marital conflicts were highly correlated (*r* = 0.65, *p* < 0.001) and the caregiver reported somewhat more marital conflicts than intergenerational conflicts [*t*(1150) = 6.70, *p* < 0.001, *g* = 0.17]. For the moderator, regional pandemic risk was associated with more educational expenditure, more frequent engagement in home learning activities, and more paternal child care time. However, regional pandemic risk was not related to maternal and paternal psychological and physical well-being before and during the pandemic. It was weakly related to more intergenerational and marital conflicts.

**Table 2:** Means (*M*), standard deviations (*SD*), and correlations for variables of interest.

	1	2	3	4	5	6	7	8	9	*N*	*M*	*SD*
**Outcomes**												
1. Educational expenditure 2020										1,155	4312.81	7298.35
2. Home learning activities 2020	0.21^**^									1,151	2.88	0.89
3. M child care time per day 2020	−0.05	0.06								485	2.96	1.48
4. M child care time per week 2020	−0.03	0.04	0.77^**^							490	34.85	36.28
5. F child care time per day 2020	0.08	0.09	0.06	0.04						419	1.45	0.97
6. F child care time per week 2020	0.03	0.04	0.06	0.06	0.82^**^					423	9.02	9.78
**Mediators**												
7. Intergenerational conflicts 2020	0.09^**^	0.06^*^	0.05	0.03	0.05	−0.00				1,154	1.49	0.82
8. Marital conflicts 2020	0.09^**^	0.04	0.11^*^	0.05	0.05	0.01	0.65^**^			1,151	1.64	0.90
**Moderator**												
9. Regional pandemic risk 2020	0.20^**^	0.07^*^	−0.00	0.04	0.07	0.12^*^	0.06^*^	0.08^**^		1,154	42.23	72.97
**Predictors**												
10. M depression 2018	−0.07	−0.04	0.07	0.09	0.00	−0.01	0.05	0.08^*^	−0.04	688	13.57	3.21
11. M psychological well-being 2018	−0.02	0.11^**^	0.03	−0.01	0.09	−0.01	−0.03	−0.06	0.00	689	0.00	1.00
12. M physical illness 2018	0.10^**^	−0.03	−0.03	−0.04	0.05	−0.02	0.14^**^	0.12^**^	0.05	689	0.01	1.00
13. M physical health 2018	0.19^**^	0.09^*^	−0.07	−0.08	−0.03	−0.05	0.01	0.05	0.06	693	1.69	0.68
14. M depression 2020	0.01	−0.10^*^	0.03	0.03	−0.05	−0.05	0.08	0.08	−0.04	666	13.57	3.67
15. M psychological well-being 2020	−0.00	0.12^**^	−0.01	−0.06	0.02	−0.01	−0.07	−0.06	0.01	664	−0.00	1.00
16. M physical illness 2020	0.11^*^	−0.06	−0.02	0.01	0.02	−0.03	0.07	0.08^*^	0.04	664	−0.00	1.00
17. M physical health 2020	0.16^**^	0.15^**^	−0.10^*^	−0.11^*^	0.04	0.06	−0.01	0.04	0.03	666	1.67	0.62
18. F depression 2018	−0.02	−0.06	0.10*	0.02	−0.12^*^	−0.17^**^	0.01	0.00	−0.05	603	12.87	3.15
19. F psychological well-being 2018	0.02	0.11^**^	0.05	0.09	0.08	0.10	−0.01	−0.07	0.00	603	0.01	0.99
20. F physical illness 2018	0.01	−0.06	−0.05	0.01	−0.11^*^	−0.09	−0.01	0.01	0.04	603	0.00	1.00
21. F physical health 2018	0.06	0.12^**^	−0.04	−0.03	0.15^**^	0.13^*^	0.03	0.03	0.04	610	1.72	0.71
22. F depression 2020	0.01	−0.06	0.04	0.05	−0.13^**^	−0.15^**^	0.02	0.07	−0.03	592	12.44	4.93
23. F psychological well-being 2020	−0.03	0.14^**^	0.07	0.02	0.13^**^	0.13^**^	−0.09^*^	−0.11^**^	0.02	578	−0.00	1.00
24. F physical illness 2020	0.02	−0.09^*^	0.03	−0.02	−0.04	−0.03	0.13^**^	0.15^**^	0.02	575	−0.00	1.00
25. F physical health 2020	0.02	0.07	0.03	0.06	−0.02	−0.02	0.03	0.04	−0.03	592	1.62	0.70

^*^p < 0.05; ^**^p < 0.01. M, Maternal; F, Paternal.

**Table 3:** Correlations of maternal and paternal psychological and physical well-being before and during the pandemic.

	1	2	3	4	5	6	7	8
1. Depression 2018		−0.34^**^	0.26^**^	−0.08	0.39^**^	−0.26^**^	0.14^**^	−0.05
2. Psychological well-being 2018	−0.30^**^		−0.30^**^	0.04	−0.40^**^	0.58^**^	−0.24^**^	−0.01
3. Physical illness 2018	0.26^**^	−0.17^**^		−0.04	0.19^**^	−0.21^**^	0.44^**^	0.05
4. Physical health 2018	−0.02	−0.02	−0.05		−0.01	0.05	0.05	0.25^**^
5. Depression 2020	0.31^**^	−0.23^**^	0.16^**^	−0.09^*^		−0.47^**^	0.24^**^	0.19^**^
6. Psychological well-being 2020	−0.16^**^	0.49^**^	−0.24^**^	−0.03	−0.37^**^		−0.31^**^	0.02
7. Physical illness 2020	0.17^**^	−0.14^*^	0.40^**^	0.02	0.26^**^	−0.27^**^		−0.00
8. Physical health 2020	−0.10^*^	0.04	0.04	0.19^**^	−0.07	−0.05	0.04	

^*^p < 0.05; ^**^p < 0.01. Above the diagonal (upper right) shows the correlations of paternal psychological and physical well-being and below the diagonal (lower left) shows the correlations of maternal psychological and physical well-being.

In Table 3, small- to medium-size autocorrelations are found for maternal and paternal depression, psychological well-being, physical illness, and physical health from before to during the pandemic. This means those with pre-existing mental health problems and large physical symptom load continued suffering from compromised well-being during the pandemic. Turning to mean-level differences before and during the pandemic in parental well-being, no mean-level differences were found for maternal [*t*(604) = 0.48, *p* = 0.63] and paternal [*t*(495) = −0.30, *p* = 0.77] depression. Possibly, this is because the 2020 assessment was conducted in the time of a low-transmission rate of COVID-19 in China. Regarding the indicators of psychological well-being, mothers reported a small decrease in their confidence in the future from before (*M* = 4.24, *SD* = 0.76) to during the pandemic (*M* = 4.14, *SD* = 0.81) [*t*(603) = −2.55, *p* = 0.01, *g* = 0.13]. Fathers reported lower satisfaction with social relationships during the pandemic (*M* = 6.94, *SD* = 1.63) as compared to before the pandemic (*M* = 7.11, *SD* = 1.53) [*t*(492) = −2.06, *p* = 0.04, *g* = 0.11]. Concerning physical illness, mothers were slightly more likely to be diagnosed with chronic diseases during the COVID-19 pandemic as compared to before (Wilcoxon signed-ranks test, *Z* = 1.98, *p* < 0.05). Regarding physical health, fathers displayed fewer health behaviors and healthy habits during the pandemic compared to before the pandemic (*Z* = −2.18, *p* = 0.03).

### 3.2. Moderated mediation models

We next tested whether the relative changes in parental well-being from 2018 to 2020 predict parental provisions of early learning and care at home after controlling for covariates (step 1 in Figure 1). The baseline model for maternal data yielded acceptable model fit (*χ*^2^(48) = 136.11, *p* < 0.001, CFI = 0.96, RMSEA = 0.04, 90% CI = [0.03, 0.05]). The results showed that improvements in maternal physical health predicted more family educational expenditure (*β* = 0.09, *p* < 0.001). Improvements in maternal psychological well-being predicted more frequent engagement in home learning activities (*β* = 0.08, *p* = 0.04). When maternal physical health deteriorated, mothers spent more time on child care per week (*β* = −0.10, *p* = 0.04). The other associations between changes in maternal well-being and provisions of early learning and care at home were not significant.

The baseline model for paternal data resulted in good model fit (*χ*^2^(48) = 66.04, *p* = 0.04, CFI = 0.99, RMSEA = 0.02, 90% CI = [0.00, 0.03]). Improvements in paternal psychological well-being predicted more frequent engagement in home learning activities (*β* = 0.13, *p* = 0.01). When paternal depressive symptoms increased, fathers spent less time on child care per week (*β* = −0.16, *p* = 0.03). In contrast, when paternal psychological well-being improved, fathers spent more time on child care per week (*β* = 0.10, *p* = 0.04). The other associations between changes in paternal well-being and provisions of early learning and care at home were not significant.

In the second step (see Figure 1), we entered intergenerational conflicts and marital conflicts into the model to test their mediating effects together with examining the direct associations between regional pandemic risk and parental provisions of early learning and care at home. Because intergenerational and marital conflicts were highly correlated, a latent variable, family conflicts, was estimated in the mediation models to account for the covariance between these two mediators. The model fit of the mediation model for maternal data was good (*χ*^2^(92) = 209.39, *p* < 0.001, CFI = 0.96, RMSEA = 0.03, 90% CI = [0.03, 0.04]). Specifically, mothers with severe physical illness before the pandemic reported more family conflicts during the pandemic (*β* = 0.14, *p* = 0.02) which, in turn, was further linked with more family educational expenditure (*β* = 0.07, *p* = 0.01, effect size = 0.01, *p* = 0.13) and mothers spending more time on child care per day (*β* = 0.12, *p* = 0.03, effect size = 0.02, *p* = 0.06). Given the non-significant effect size of these two indirect paths, these results should only be viewed as possibilities. Moreover, higher regional pandemic risk predicted more family educational expenditure (*β* = 0.13, *p* < 0.01).

The mediation model for paternal data yielded good model fit as well (*χ*^2^(92) = 143.57, *p* < 0.001, CFI = 0.98, RMSEA = 0.02, 90% CI = [0.02, 0.03]). However, none of the indirect paths was significant. Again, family conflicts were predictive of more family educational expenditure (*β* = 0.07, *p* = 0.02). Regional pandemic risk also predicted more family educational expenditure (*β* = 0.14, *p* < 0.01). Furthermore, there was a trend for a positive association between regional pandemic risk and the time fathers spent on child care per week (*β* = 0.09, *p* = 0.05). It is noteworthy though that after entering the mediators and moderator, the direct associations with the time fathers spent on child care per week were attenuated for the change in paternal depression (*β* = −0.15, *p* = 0.05) and the change in paternal psychological well-being (*β* = 0.09, *p* = 0.07).

Considering the large number of parameters estimated, which may limit the statistical power to detect effects, we adjusted the mediation models by removing non-significant correlations of covariates with parental well-being in 2018 and parental provisions of early learning and care at home in 2020 and double-checked the robustness of the results. The parsimonious models did not differ from the original mediation models for maternal data [Δ*χ*^2^(45) = 47.19, *p* = 0.38] and paternal data [Δ*χ*^2^(38) = 37.67, *p* = 0.48]. Our results noted above were robust and did not change. Based on the parsimonious mediation models, we established the moderated mediation models (Step 3 in Figure 1) to test the moderating effects of regional pandemic risk on the significant associations between changes in parental well-being and parental provisions of early learning and care and between family conflicts and parental provisions of early learning and care. As we included latent interaction terms in the models, model fit indices including *χ*^2^, CFI, and RMSEA, were no longer available.

**Figure 2:**
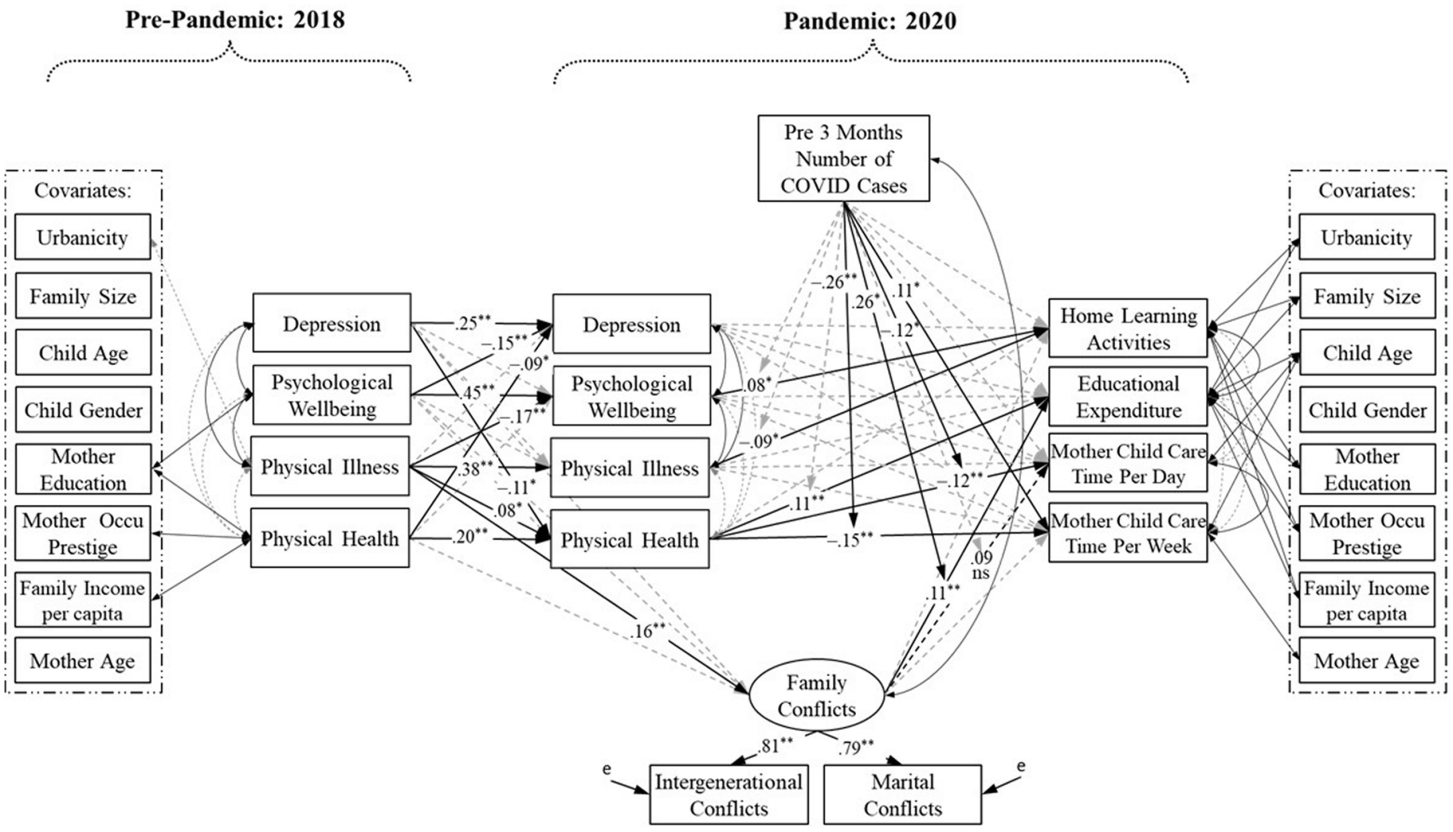
Moderated mediation model for maternal data. ^*^p < 0.05; ^**^p < 0.01. Occu = Occupational.

In the final moderated mediation model for maternal data (see Figure 2), we found three significant moderations. Regional pandemic risk strengthened the negative association between the change in maternal physical health and the time mothers spent on child care per day (*β* = −0.12, *p* = 0.04) as well as the negative association between the change in maternal physical health and the time mothers spent on child care per week (*β* = −0.26, *p* < 0.01). Further probing of these two moderations with the simple slope technique revealed a similar pattern. For mothers in the provinces with no cases reported in the past 3 months (low-risk regions), the change in physical health was not related to the time mothers spent on child care. For mothers in the provinces with 25 cases reported in the past 3 months (medium-risk regions), negative changes in physical health predicted mothers spending longer on child care per day (*B* = −0.22, *SE* = 0.10, *p* = 0.03) and per week (*B* = −5.74, *SE* = 2.45, *p* = 0.02). Similarly for mothers in the provinces with 65 cases reported in the past 3 months (high-risk regions), negative changes in physical health predicted mothers spending longer on child care per day (*B* = −0.37, *SE* = 0.12, *p* < 0.01) and per week (*B* = −14.30, *SE* = 3.82, *p* < 0.01). These results indicate that regional pandemic risk may amplify the negative impacts of worsened physical health, further trapping mothers in heavier responsibilities of child care.

Moreover, the positive association between family conflicts and family educational expenditure was strengthened by regional pandemic risk (*β* = 0.28, *p* = 0.02). Follow-up simple slope analyses showed that for mothers in low- and medium-risk regions, family conflicts were not related to family educational expenditure. In contrast, for mothers in high-risk regions, we found a positive link between family conflicts and family educational expenditure (*B* = 1.31, *SE* = 0.52, *p* = 0.01). Of note, the link between family conflicts and the time mothers spent on child care per day was attenuated and no longer significant after including the interaction term (*β* = 0.09, *p* = 0.08). However, regional pandemic risk was directly related to mothers spending longer on child care per week (*β* = 0.11, *p* = 0.04).

**Figure 3:**
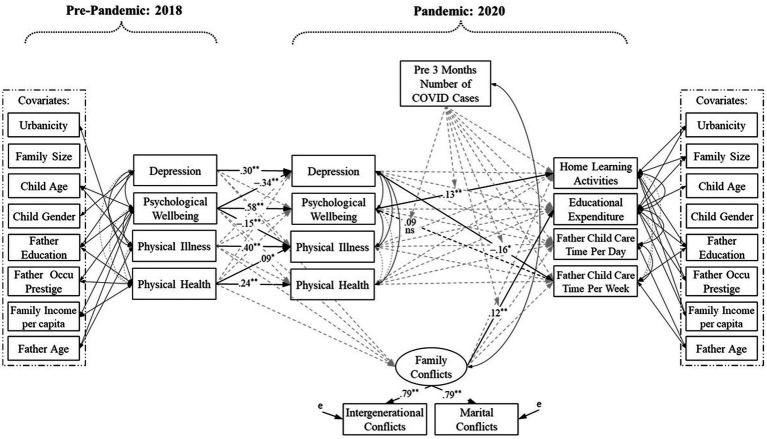
Moderated mediation model for paternal data. ^*^p < 0.05; ^**^p < 0.01. Occu = Occupational.

In the moderated mediation model for paternal data (see Figure 3), none of the moderations of regional pandemic risk was significant. However, there was a trend for its moderating effect on the association between family conflicts and family educational expenditure (*β* = 0.27, *p* = 0.06). Yet the simple slope analyses revealed that, though the strengths differed, for fathers in low- (*B* = 0.65, *SE* = 0.27, *p* = 0.02), medium- (*B* = 0.77, *SE* = 0.32, *p* = 0.02), and high-risk regions (*B* = 0.94, *SE* = 0.39, *p* = 0.02), family conflicts were all related to more family educational expenditure.

## 4. Discussion

The COVID-19 pandemic has profoundly impacted family functioning and children’s early learning across the world. Extant evidence indicates that the COVID-19 pandemic had a negative impact on families. However, in high-resource contexts, socially advantaged families reported positive impacts such as more time spent with the family, less time needed to commute to work, and less work-related stress (e.g., Lateef et al., 2021). Without adequate support, most parents with young children experienced stress, and more research is needed to inform evidence-based interventions and policies that may mitigate parental health consequences to promote nurturing care and early learning at home during this unprecedented time (Brock and Laifer, 2020; Roubinov et al., 2020; Wang et al., 2020). The current study examined how family investment in the early learning and care young Chinese children received at home was sculpted by the COVID-19 pandemic, potentially through parental psychological and physical well-being and family relationships.

Using a large longitudinal sample of Chinese mothers and fathers with young children, we found that the changes in maternal psychological and physical well-being and the changes in paternal psychological well-being were associated with family educational expenditure, home learning activities, and the time parents spent on child care. Overall, indicators of maternal well-being were more relevant to the provisions of early learning and care as compared with the same indicators of paternal well-being. Notably, we found preliminary evidence of a moderated mediation path from pre-existing maternal physical illness to more family educational expenditure amid the pandemic through family conflicts, and this indirect path was pronounced only for mothers in regions with a relatively high-transmission risk of COVID-19.

### 4.1. Parental psychological and physical responses to the COVID-19 pandemic

Our first aim was to examine differences in parental well-being before and during the pandemic and, second, elucidate how these changes may relate to parental provisions of early learning and care at home. Decreased psychological well-being, physical health, and elevated physical symptom load were found in Chinese parents with young children. The results on reductions in parental psychological well-being are consistent with the previous literature (see Lateef et al., 2021, for a review). Our findings concerning an increased risk of chronic disease for mothers and lowered physical health for fathers extend the knowledge base on the detrimental impacts of the pandemic on parents specifically. However, these mean-level changes were modest in extent, and we did not find mean-level changes in maternal and paternal depression. Such a result should be understood in light of the development of the pandemic situation. The 2020 assessment was conducted when the pandemic was largely under control in China. Because life has gone back to normal in most places, the well-being of Chinese parents might have rebounded from the troughs experienced after the initial outbreak of COVID-19.

Yet we also note that this parental resilience should not be taken for granted (see also Prime et al., 2020; Rao and Fisher, 2021). The stable autocorrelations of parental depression, psychological well-being, physical illness, and physical health from the pre-pandemic to pandemic times indicate that those with pre-existing mental health problems and physical symptoms adapted poorly to pandemic-related social disruptions (Roubinov et al., 2020; Yoshikawa et al., 2020; Robinson et al., 2022). Alarmingly, our results revealed that there were interrelations between psychological and physical well-being, such that pre-existing mental and physical problems may translate into the other problem during the pandemic for mothers (Figure 2) and pre-existing psychological issues may intervene in paternal physical health during the pandemic (Figure 3). These findings extend the seminal works of Brock and Laifer (2020) and Prime et al. (2020), suggesting that parental psychological responses and physical responses to the pandemic should be considered simultaneously to fully apprehend how parents behave and function in this unique situation.

Further consolidating our argument, results from the path models, conducted to examine the second aim of our research, showed that, for mothers, improvements in physical health predicted more family educational expenditure and increases in physical illness predicted less frequent engagement in home learning activities. The effects exerted by maternal physical well-being were shown after accounting for the influences exerted by the changes in maternal psychological well-being. This suggests that maternal physical well-being determines the monetary and non-monetary investment a family could provide for home-based learning and homeschooling during the COVID-19 times. In other words, when mothers had better physical health and less physical illness, they could financially contribute to educating the young child by buying books, toys, and e-learning facilities and be frequently involved in home learning activities with the young child.

Moreover, congruent with other studies (Lee et al., 2021; Treviño et al., 2021), maternal and paternal psychological well-being improvements predicted more frequent engagement in home learning activities, and increases in paternal depressive symptoms predicted fathers spending less time on child care per week. These findings are expected because both theories (Prime et al., 2020) and empirical research (e.g., Peacock-Chambers et al., 2017; Nuttall et al., 2019) have suggested that life satisfaction and self-efficacy may motivate parents to put effort in and make time to provide nurturing care and create a home learning environment. In contrast, depressive symptoms may limit active participation in parent–child interactions and communications. Therefore, our findings add more evidence to the relevant literature by confirming these processes with Chinese parents with young children and during uncharacteristic times such as the COVID-19 pandemic.

### 4.2. Mediations of family conflicts and moderations of regional pandemic risk

Recognizing the complexity of pandemic-related consequences and influence processes (Brock and Laifer, 2020; Roubinov et al., 2020), we tested the mediation of family conflicts in the associations between parental well-being before the pandemic and parental provisions of early learning and care. There was little evidence to support such indirect paths, except for family conflicts bridging the link between maternal physical illness in 2018 and family educational expenditure in 2020. As this indirect effect was not significant (even in the parsimonious mediation model), we are cautious about the existence of this mediation across all parent populations. We assume that family conflicts, including intergenerational and marital conflicts, are much more likely to occur in families undergoing home confinement relative to other families (Wang et al., 2020). To some extent, frequent family conflicts reflect immediate reactions from family systems toward pandemic-related social disruptions, which might not be that relevant to parental well-being assessed 2 years before the pandemic.

Notwithstanding the weak mediation of family conflicts, robust moderating effects of regional pandemic risk were found on the associations between the change in maternal physical health and the time mothers spent on child care and the associations between family conflicts and family educational expenditure. These results were trustworthy because, for the first moderating effect, a similar pattern was replicated for the time mothers spent on child care per day and per week; for the second moderating effect, the result was at least replicated for both mothers and fathers from high-risk regions. Hence, these findings provide nuanced knowledge of how pandemic-related factors penetrate normal family functions and affect parental provisions of early learning and care at home.

Specifically, only for mothers in medium- and high-risk regions did the decrease in physical health from the pre-pandemic to pandemic predict mothers spending longer on child care. Indeed, as documented in a qualitative study, mothers expressed that they were physically drained and fully occupied by the responsibilities of child care and work during the pandemic (Weaver and Swank, 2021). A possible interpretation is that mothers living in medium- and high-risk regions needed their children to stay at home because child care services and preschools were unavailable. Consequently, these mothers could not participate in exercise but had to spend most of their time caring for their children. Maternal sleep habits may also be disturbed, resulting from changes in everyday routines caused by home quarantine and lockdowns.

Furthermore, the association between family conflicts and family educational expenditure was significant for parents in high-risk regions. Possibly, families in high-risk regions had to spend money to compensate for the loss of early learning due to preschool closures, albeit already struggling with financial constraints. These families, therefore, had a high likelihood of family conflicts. For fathers in low- and medium-risk regions, family conflicts were still associated with more educational expenditure, yet this was no longer the case for mothers in these regions. A possible interpretation lies in gender roles in Chinese culture that view fathers as the breadwinner who is responsible for earning money to cover the cost of the child’s education. Relatedly, financial hardships caused by the pandemic might impede not only parental, especially paternal, investment in children’s early learning but are also likely lead to family conflicts and arguments in the matter of the allocation of family resources during the COVID-19 pandemic (e.g., saving money for medical costs of a family member). This finding is particularly relevant to the whole-family-function model proposed by Prime et al. (2020). The COVID-19 pandemic affected multiple aspects of the family system. Disturbance in any subsystem may spill over to influence other family subsystems.

### 4.3. Strengths, limitations, and implications

This research has several strengths. First, we used longitudinal instead of cross-sectional data collected before and during the pandemic to demonstrate how parents psychologically and physically respond to the pandemic and its relevant confinement measures. Second, we included not only parental aversive responses (depression and physical illness) but also positive indicators of parental well-being (psychological well-being and physical health) as predictors, which enables us to provide a comprehensive snapshot of how changes in parental well-being affect their provisions of early learning and care at home. Third, we considered the complexity of pandemic-related mechanisms and examined both mediation processes and moderation processes in the study.

This study also has limitations. First, there is a lack of child outcome variables, which limits the possibility of examining whether and how parental provisions of early learning and care at home further forecast early childhood development. With available data, future research may consider investigating whether the associations of family educational expenditure, home learning environment, and the time parents spent on child care with preschoolers’ cognitive, social, and emotional outcomes differ before and during the pandemic owing to altered settings of early childhood education and care. Second, although the sample size was large, some variables had missing data, which inevitably caused bias in model estimation. Third, we could not extract demographic information, especially family income and job status, at the 2020 assessment of the CFPS due to data unavailability currently. As a result, we could not estimate social disruptions at the family level and test some of our explanations. Future studies may incorporate these family-level factors to replicate our findings.

The results of this study have other important theoretical and practical implications. First, from another perspective, we support the assumption of a “gendered pandemic” (Petts et al., 2021; Yavorsky et al., 2021). Despite moderate mean-level changes in well-being for both mothers and fathers, the changes in maternal psychological and physical well-being were more strongly associated with provisions of early learning and care at home relative to the changes in paternal well-being. This finding highlights gendered roles in providing child care and homeschooling, especially in early childhood. Notably, regional pandemic risk mainly moderated significant associations for maternal data. Higher levels of regional pandemic risk directly predicted mothers spending longer on child care, but not the time fathers spent on child care, after accounting for covariates and covariance with other variables. These results underscore the unequal caregiving burden put on mothers during the pandemic (Augustine and Prickett, 2022). Given these observed gender inequalities in provisions of home-based education and child care, policies that aim at mitigating gender gaps in responsibilities of child and family care (e.g., father’s child care leave) and alleviating maternal stress and pressure (e.g., emergency residential child care services) during the pandemic are warranted.

Moreover, our findings evince that parental psychological and physical well-being are related to their provisions of early learning and care. While we recognize that the Prime et al. (2020) model has laid the cornerstone for understanding how the COVID-19 pandemic impacts family functioning, as suggested by Roubinov et al. (2020), researchers and practitioners need to take the complexity of pandemic-induced consequences into account. In the latter stages of the COVID-19 pandemic, governments had implemented various confinement measures. Such regulations immediately led to changes in daily routines. A more likely scenario is that parental lifestyles and healthy habits (i.e., physical health) were altered first, which in turn, generated unfavorable psychological responses during the lockdown and quarantine times. To grasp the nature of these processes, a biopsychosocial model should be considered to better appreciate the all-embracing impacts of the pandemic on parental well-being and on associations between parental well-being and children’s early learning.

## 5. Conclusion

In conclusion, drawn upon a large longitudinal sample of Chinese families with young children, the current study demonstrates that parental psychological and physical well-being deteriorated slightly from pre-pandemic levels during the pandemic. Changes in parental psychological and physical well-being were associated with parental provisions of early learning and care at home amid the COVID-19 pandemic. There were stronger associations between maternal psychological and physical well-being and home-based early care and education for young children than between paternal well-being and home-based care and education for young children. Moreover, family conflicts were positively associated with concurrent family educational expenditure. This phenomenon is interpreted in light of traditional values related to gender roles and unexpected events such as the pandemic. Regional pandemic risk exacerbated family arguments in the matter of allocation of family resources in the face of the unexpected crisis. Taken together, our findings reveal that biopsychosocial indicators of parental well-being, the quality of relationships between members of the family, and the regional context are all important factors for understanding parental provisions of early learning and care at home during the current COVID-19 pandemic.

## Data availability statement

The raw data supporting the conclusions of this article will be made available by the authors, without undue reservation.

## Ethics statement

Ethical review and approval was not required for the study on human participants in accordance with the local legislation and institutional requirements. The patients/participants provided their written informed consent to participate in this study.

## Author contributions

SD and NR designed the study. SD extracted and analyzed the data and drafted the manuscript. NR made essential comments and substantial revisions to the manuscript. All authors contributed to the article and approved the submitted version.

## Funding

This work was supported by two grants from the Research Grants Council (RGC) Hong Kong SAR, China, including Postdoctoral Fellowship PDFS2223-7H04 to SD and General Research Fund HKU 17609521 to NR; and a UKRI Collective Fund Award (ES/T003936/1), UKRI GCRF (PI: Alan Stein).

## Conflict of interest

The authors declare that the research was conducted in the absence of any commercial or financial relationships that could be construed as a potential conflict of interest.

## Publisher’s note

All claims expressed in this article are solely those of the authors and do not necessarily represent those of their affiliated organizations, or those of the publisher, the editors and the reviewers. Any product that may be evaluated in this article, or claim that may be made by its manufacturer, is not guaranteed or endorsed by the publisher.
